# Wild food plants used by the Tibetans of Gongba Valley (Zhouqu county, Gansu, China)

**DOI:** 10.1186/1746-4269-10-20

**Published:** 2014-02-06

**Authors:** Yongxiang Kang, Łukasz Łuczaj, Jin Kang, Fu Wang, Jiaojiao Hou, Quanping Guo

**Affiliations:** 1 College of Forestry, Northwest A&F University, 712100 Yangling, Shaanxi, PR China; 2 Department of Botany and Biotechnology of Economic Plants, Institute of Applied Biotechnology and Basic Sciences, University of Rzeszów, Werynia 502, 36-100 Kolbuszowa, Poland; 3 Forestry Academy of Bailongjiang Forestry Administration Bureau, 746010 Liangshui, Gansu, PR China

**Keywords:** Wild edible plants, Brugchu, Ethnobotany, Ethnomycology, Caltha palustris, Lactic fermentation, Lacto-fermented

## Abstract

**Background:**

The ethnobotany of Tibetans is a seriously under-studied topic. The aim of the study was to investigate knowledge and use of wild food plants in a valley inhabited by Tibetans in the Gannan Tibetan Autonomous Region.

**Methods:**

The field research was carried out in a wooded mountain valley in 9 neighbouring villages the Zhouqu (Brugchu) county, and comprised 17 interviews with single informants and 14 group interviews, involving 122 people altogether.

**Results:**

We recorded the use of 81 species of vascular plants from 41 families. Fruits formed the largest category, with 42 species, larger than the wild greens category, with 36 species. We also recorded the culinary use of 5 species of edible flowers, 7 species with underground edible organs and 5 taxa of fungi. On average, 16.2 edible taxa were listed per interview (median – 16). Green vegetables formed the largest category of wild foods (mean – 8.7 species, median – 9 species), but fruits were listed nearly as frequently (mean – 6.9, median – 6). Other categories were rarely mentioned: flowers (mean – 0.2, median – 0), underground edible parts (mean – 0.3, median – 0) and mushrooms (mean – 1.5, – median 1).

Wild vegetables are usually boiled and/or fried and served as side-dishes (*cai*). They are often lacto-fermented. Wild fruits are mainly collected by children and eaten raw, they are not stored for further use. The most widely used wild vegetables are: *Eleuterococcus* spp., *Pteridium aquilinum*, *Helwingia japonica, Aralia chinensis, Allium victorialis, Pteridium aquilinum, Ixeris chinensis*, *Thlaspi arvense* and *Chenopodium album.* The culinary use of *Caltha palustris* as a green vegetable is very interesting. In its raw state, marsh marigold is a toxic plant, due to the presence of protoanemonin. In this area it is dried or lactofermented before use. The most commonly eaten fruits are: *Pyrus xerophila*, *Prunus salicina*, *Berchemia sinica*, *Rubus* spp. and *Eleagnus umbellata.*

**Conclusions:**

The number of wild taxa eaten in the studied valley is relatively large compared to most studies from around the world. However, compared to the northern slope of the Qinling, in Shaanxi, the list is considerably shorter, in spite of the similar methodology applied and similar research effort involved.

## Background

Wild food plants still function as an important part of human nutrition in many parts of the globe. Although nowadays they usually provide only a minor proportion of daily calories, they are important sources of vitamins, microelements and other health supporting chemicals. They have always enabled human survival in times of crises [1-5]. The diversity of wild food plants consumed depends mainly on which species are available in sufficient quantity in the surrounding vegetation, the socioeconomic status of the population and cultural attitudes towards different categories of wild food. China is a very interesting arena for studies on wild food use traditions, as in all three above-mentioned aspects there are conditions that support the wide use of plants [6]. It contains an immense diversity of habitats and possesses numerous biodiversity hotspots. Some of its populations in remote rural areas still have a relatively low economic status, which binds people to traditional subsistence agriculture and exploitation of natural resources. Moreover, the rural population exploits different categories of wild food, including numerous species of wild vegetables, and is in itself diverse, consisting of many ethnic minorities, which differ in their plant utilization habits.

The ethnobotany research in China, concerning wild food plants, has so far been concentrated mainly on Yunnan [7-12] and Inner Mongolia [13,14], also as far as wild food is concerned. Although some papers on the use of wild food plants have been published from other parts of China (e.g. Sichuan [15,16], Hunan [17] etc.), some provinces are seriously under-studied ethnobotanically. An example of such a place is the province of Gansu, for which we found only one small article on wild vegetables, and only for the easternmost part of the province inhabited by Han Chinese [18]. Gansu is an ethnic mosaic of a few ethnic groups: Han Chinese, Hui and Tibetans [19]. In spite of the tremendous cultural diversity of different Tibetan ethnic groups and their diverse plant use, there is relatively little ethnobotanical literature devoted to the Tibetan people in China [12,20-22]. There is a particularly scarce ethnobotanical literature concerning the use of wild food plants by Tibetans, also in other provinces, with only one major article on the wild food uses of the Tibetans in Shangri-La (NW Yunnan) [12].

Although Tibetans are classified in China as one minority (族藏, Z*angzu*), they consist of a mosaic of several Tibetan languages, dialects and ways of life, with six main groups/dialects, out of which three occur in China classified as Ü-Tsang, Kham and Amdo [23]. The linguistic situation on the Gansu – Sichuan border is little studied, and it is one of the regions in which ever newer dialects are described (e.g. [24]). Some linguists distinguish over twenty separate distinct languages in the Tibetan group of languages [25]. As Nicolas Tournandre puts it: “there are 220 ‘Tibetan dialects’ derived from Old Tibetan and nowadays spread across 5 countries: China, India, Bhutan, Nepal and Pakistan which may be classed within 25 dialect groups, i.e. groups which do not allow mutual intelligibility. (…) Thus if we set aside the notion of standardization, I believe it would be more appropriate to speak of 25 languages derived from Old Tibetan. This is not only a terminological issue but it gives an entirely different perception of the range of variation. When we refer to 25 languages, we make clear that we are dealing with a family comparable in size to the Romance family which has 19 groups of dialects”.

To fill the gap in the ethnobotanical exploration of the northwestern part of China we aimed at documenting the use of wild food plants in one Tibetan valley in SW Gansu, on the edge of the Qinling Mountains and the Tibetan Plateau. As we have been, since 2011, carrying out similar research in other parts of the Qinling, inhabited by Han Chinese, one of the research questions also compared the pattern of wild food plant use with our previous studies [26-28].

## Methods

### Study site

**Figure 1:**
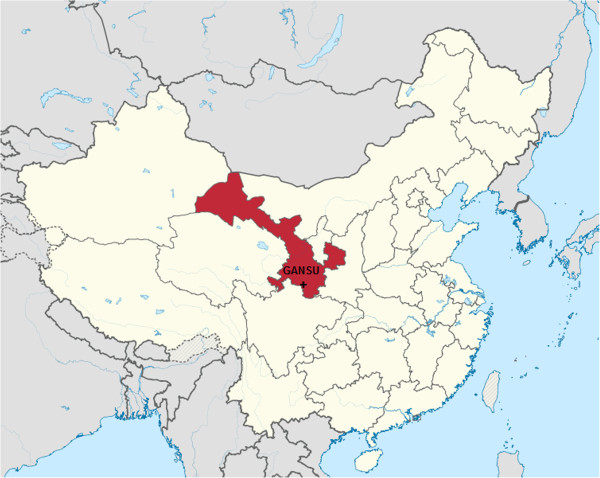
The location of the study area in China.

The province of Gansu is located in northwest China, bordering Xinjiang and Qinhai in the west, Ningxia and Inner Mongolia in the north, Sichuan in the south and Shaanxi in the east (Figure 1). The vegetation changes from desert in the north and centre through dry grasslands to deciduous forests in the mountainous southern Gansu. In the south-west of Gansu, the Gannan Tibetan Autonomous Region was created where the Tibetan minority constitutes a large part of the population. Both the Chinese and Tibetans have a long history of settlement in this area as the region has formed a natural ethnic border between the Han Chinese and Tibetan speaking peoples [18].

Tibetans in Gannan constitute a very diverse collection of subsistence economies and speak a variety of dialects. In the northern part of the territory, in the grasslands, animal herders predominate. The Diebu (Tewo) Tibetans, although living among forests, live mainly from animal husbandry, whereas in the southern part of the region we studied, in some forested valleys, plant cultivation is the main source of subsistence.

**Figure 2:**
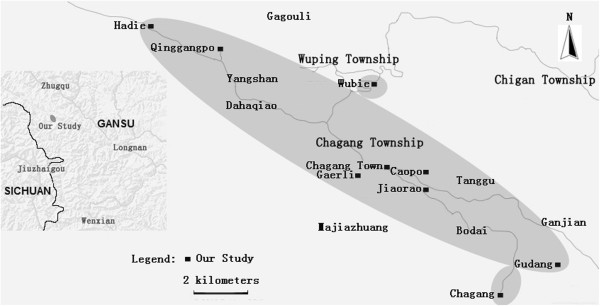
The map of the studied area.

**Figure 3:**
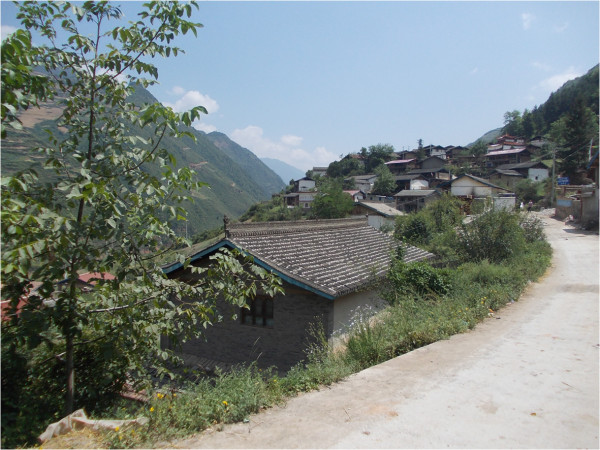
All the Tibetan villages in the valley are tight groups of homesteads located on hilltops.

**Figure 4:**
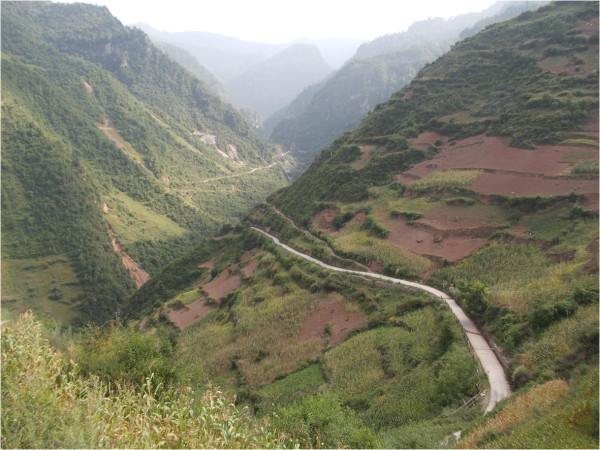
The landscape of Gongba valley around Chagang.

The studied valley is located along a small river, Gongba, which is a tributary of Bailongjiiang (White Dragon River). Bailongjiang valley and its surrounding areas constitute a mountainous forefront of the Tibetan Plateau. We studied most Tibetan villages in two townships (镇 *zhen*): Chagang and Wuping [29]. The two townships are located in the southwestern part of the Zhouqu country (Brugchu or Zhugqu in Tibetan), beside the upper Gongba River basin, separated by massive mountain ranges both from the main part of Zhouqu country in the east and the Sichuan province in the southwest (Figures 2, 3 and 4).

The Chagang Township has an area of 157 km^2^. The average annual rainfall is 680 mm (mainly in summer months), the annual average temperature is 9.1°C, and the frost-free period lasts 220 days. The average annual net income is 1376 Yuan. The total population is 3624, of which farmers account for 3197. There are Tibetan, Han and Hui in the township, but the Tibetan population accounts for 83% of the total population [29].

Wuping township is located upstream from the Chagang township. It has an area of (429 km^2^). The annual average temperature ranges between 6°C and 12°C, the frost-free period lasts 160 to 200 days. It consists of 10 administrative villages, including 32 small settlements, with a total population of 6962. There are 6240 farmers, including 2889 Tibetans, who constitute 41.5% of the total population [29].

The studied villages are purely Tibetan and usually set at the top of a hill, with a few dozen houses, all very close to each other, e.g. Jiaorao Village, with 83 households and 483 inhabitants, Gudang – 62 houses, 300 people, Gaerli – over 500 people and Hadie – 116 houses, 618 inhabitants [29]. We studied nine villages altogether, in Chagangzhen township (插岗镇): Jiaorao (角绕), Jiaoerli (噶儿里), Caopo (草坡), Qinggangpo (青岗坡), Chagang (插岗村), Gudang (古当), Wubie (吾别); in Wupingzhen township (武坪镇): Hadie (哈迭) and Wuping (武坪). The altitude ranged from 1782 to 2140 m a.s.l.

The studied population consists of subsistence farmers cultivating barley, potatoes, maize, flax, buckwheat, broad bean, pea, rape-seed and some fruit trees, e.g. apple, pear, walnuts and Sichuan pepper (*Zanthoxylum bungeanum*). More rarely fungi (mainly *Auricularia* and *Grifola umbellata*) and medicinal herbs (*Gastrodia, Bupleurum chinense, Codonopsis*) are cultivated.

### Data collection

The field research was conducted in August 2013 using the Rapid Rural Appraisal approach [30,31], and included 31 freelisting interviews (17 interviews with single informants and 14 group interviews), which altogether involved 122 people (70 men and 52 women).The mean age of the participants was 50 (median 48, aged from 12 to 85; 52 women and 70 men). For the seventeen people (11 men and 6 women) who were interviewed separately the mean age was 53, median 51, youngest interviewee – 23, the oldest one – 75).

**Figure 5:**
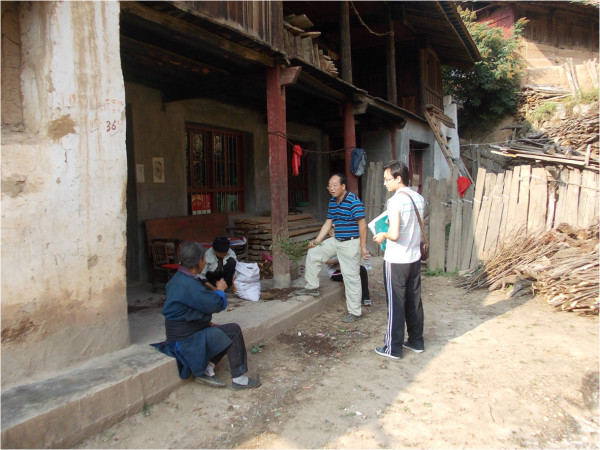
A typical two-storey house.

**Figure 6:**
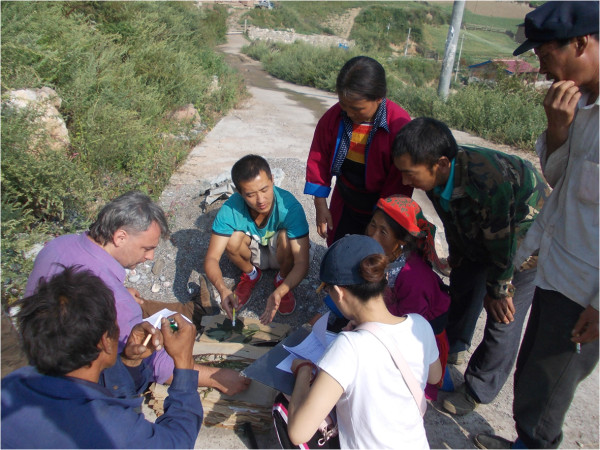
Local inhabitants eagerly took parts in interviews and specimen collection.

The listed taxa were identified using transect walks for cross-checking of the gathered herbarium specimens and semi-structured interviews with key informants (Figures 5 and 6). During freelisting we separately asked which species of wild vegetables (including underground organs), wild fruits and wild mushrooms were used. Making three separate freelists enabled the comparison of the use of these categories and helped elicit answers from the respondents, who categorized the studied wild products in the same way [32,33]. Freelists were made orally and written down on the spot by our team. The interviews were additionally recorded using a digital sound recorder. The interviews were carried out in standard Mandarin Chinese (most of the population is bilingual and attends a Chinese language school). We recorded the local Chinese language names as well as local Tibetan names and transcribed them according to the International Phonetic Alphabet. The study started from a few recommended informants, including village leaders. These people were well-known to one of the authors of the article (Quanping Guo), who is the local representative of the forestry authorities and has spent much time with local residents working in the forests for 31 years. He also authored an article on local dible mushroom resources [34]. The rest of the interviewees were found by systematic walks through the village and surrounding fields, visiting houses and asking the inhabitants if they wanted to take part in the study. Voucher specimens are stored in the Department of Forestry, Northwest A&F University in Yangling.

The research was carried out following the code of ethics of the American Anthropological Association [35] and the International Society of Ethnobiology Code of Ethics [36]. Oral prior informed consent was acquired. Statistics were calculated using the open access statistical package PAST [37,38].

## Results and discussion

**Figure 7:**
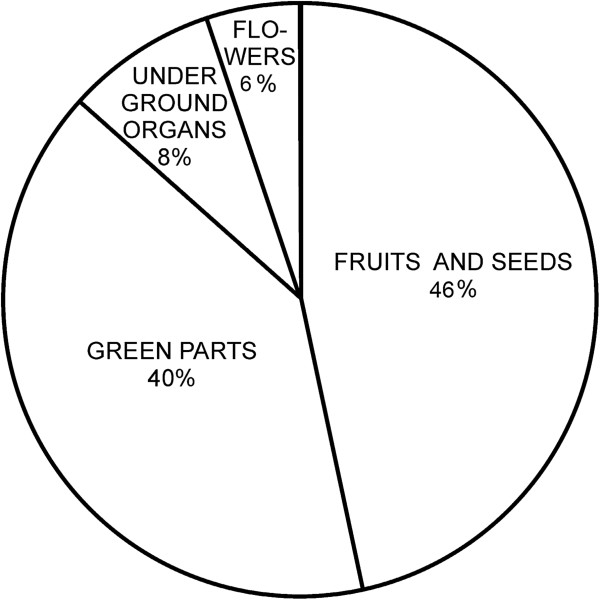
The proportion of different use categories in the list of wild food plants.

We recorded the use of 81 species of vascular plants from 41 families (Tables 1 and 2; Figure 7). The Rosaceae family was the most represented. Fruits formed the largest category, with 41 species, larger than wild greens with 36 species. We recorded the culinary use of only five species of edible flowers and seven species with underground edible organs. The use of only five fungi taxa was recorded (Table 3). Most respondents knew both the Tibetan and Chinese names.

**Table 1:** Twenty most frequently mentioned wild food plants in the freelisting questionnaire

**Species**	**Part**	**Frequency (N = 31)**
*Eleuterococcus giraldii & E. leucorrhisus*	Opening buds	30
Ferns in general (‘zhala’)	Frond stalks	30
*Helwingia japonica*	Young leaves	29
*Aralia chinensis*	Opening buds	26
*Pyrus xerophila*	Fruits	20
*Prunus salicina*	Fruits	19
*Berchemia sinica*	Fruits	17
*Allium victorialis*	Leaves	16
*Pteridium aquilinum*	Fronds	16
*Rubus mesogaeus & R. thibetanus*	Fruits	16
*Juglans cathayensis*	Kernel	14
*Thlaspi arvense*	Young aerial parts	14
*Eleagnus umbellata*	Fruits	13
*Ixeris chinensis*	Young aerial parts, roots	12
*Rubus amabilis*	Fruits	12
*Schisandra sphenanthera*	Fruits	12
*Fragaria pentaphylla*	Fruits	11
*Auricularia* sp.	Whole mushroom	10
*Chenopodium album*	Young aerial parts	10
*Decaisnea fargesii*	Fruit pulp	8
*Morchella* spp.	Whole mushroom	8
*Vitis piasezkii*	Fruits	8

**Table 2:** Wild food plants collected by at least two informants

**Family**	**Species**	**Part collected**	**Local Chinese name**	**Local Chinese name (pinyin)**	**Local Tibetan name (broad transcription of pronunciation according to IPA)**^**$**^
Actinidiaceae	*Actinidia tetramera* Maxim.	F	-	-	$$\overset{⌢}{\mathrm{dz}}\mathrm{ɨmә}\overset{⌢}{\mathrm{dz}}\mathrm{ile}$$
Amaranthaceae	*Amaranthus retroflexus* L.*	L	绿苋	Liu Xian	ɕir
	*Chenopodium album* L.	L	灰灰菜, 灰条	Hui Hui Cai, Hui Tiao	(ng)tieni
	*Acroglochin persicarioides* Moq.*	L	复灰条	Fu Hui Tiao	tieniliәmә [literally ‘sheep Chenopodium’]
Amaryllidaceae	*Allium victorialis* L.	L	鹿角菜	Luo Er Jiu	ʐe:/ʐɨ:
	*Allium* sp.	L	老葱, 野蒜, 野葱	Lao Cong, Ye Suan, Ye Cong	$$\overset{⌢}{\mathrm{dz}}\mathrm{uɔma},$$ guɔ
Anacardiaceae	*Rhus verniciflua* Stokes	L	刺菜	Ci Cai	sihei
Apiaceae	*Carum carvi* L.	F(SP)	野茴香	Ye Hui Xiang	gɨni
Araliaceae	*Eleutherococcus giraldii* (Harms) T.Nakai ,	L	五爪菜	Wu Zhua Cai	si:zana, sɨriana
	*Eleutherococcus leucorrhizus* Oliv.	L	五爪菜	Wu Zhua Cai	si:zana, sɨriana
	*Aralia chinensis* L.	L	木兰头	Mu Ian Tou	liæ(n)mə, liәmu
Asteraceae	*Artemisia subdigitata* Mattf.*	L	牛尾蒿	Niu Wei Hao	k(h)ɨeba, k(h)aba
	*Ixeris chinensis* Nakai.	L, R	苦麻菜	Ku Ma Cai	kuga, kuhua
	*Prenanthes macrophylla* Franch.*	L	山尖子	Shan Jian Zi	pahu:, paɣu:
	*Saussurea* sp.	L	筒筒菜	Tong Tong Cai	$$\overset{⌢}{\mathrm{dʑ}}\mathrm{ini}$$
	*Sinacalia tangutica* (Maxim.) B.Nord.**	R	毛鞋	Mao Hai	paibɔ, piaba, paibie
	*Sonchus oleraceus* L.	L	筒筒菜	Tong Tong Cai	wu: [mainly], (ng)wu:, muwawu:, muwawu:
	*Taraxacum* cf *mongolicum* Han.-Mzt	L, R	蒲公英	Pu Gong Ying	aukuɣua, aukəɣa
Athyriaceae	*Athyriopsis* sp.	L	白蕨	Bai Jue	$$\overset{⌢}{\mathrm{dʐ}}\mathrm{ala}$$
Brassicaceae	*Brassica campestris* L.	L	油菜	You Cai	gu, gɔu
	*Cardamine macrophylla* Willd.	L	湿根菜	Shi Gen Cai	$$\overset{⌢}{\mathrm{tɕ}}\mathrm{idi}$$
	*Thlaspi arvense* L.	L	苦根菜	Ku Gen Cai	$$\overset{⌢}{\mathrm{dʐ}}a:\left(n\right)$$
Campanulaceae	*Adenophora stricta* Miq.	L, R	泡参	Pao Shen	$$\overset{⌢}{\mathrm{dʐ}}\mathrm{әmu}\overset{⌢}{\mathrm{dʐәla}}$$
Caprifoliaceae	*Lonicera standishii* Carr.	F	荞皮子	Qiao Pi Zi	(k)alaɨala, asa, $\mathrm{kagu}\overset{⌢}{\mathrm{dʑ}}\mathrm{ie}$, kai/gai, ɕiese(ng)
	*Lonicera tragophylla* Hemsl. ex Forb. & Hemsl.	Fl	鱼鱼花	Yu Yu Hua	mudesɨbe, nɨmenu, $\overset{⌢}{\mathrm{dz}}\mathrm{ɨmutza}$:
	*Triosteum pinnatifidum* Maxim.	F	白蛋	Bai Dan	kelu, keɨü, kalɔilɔ, alaɨala
	*Triosteum himalayanum* Wall.	F	-	-	$$\overset{⌢}{\mathrm{dz}}\mathrm{ɨben},\;\overset{⌢}{\mathrm{dʑ}}\mathrm{ib}\left(i\right)\mathrm{en},\;\overset{⌢}{\mathrm{dʑ}}\mathrm{isxxxniuga},\;\overset{⌢}{\mathrm{dz}}\mathrm{ɨmasɨ},\;\mathrm{bɔ}\overset{⌢}{\mathrm{dʐ}}\mathrm{ɨma}$$
	*Viburnum betulifolium* Batalin	F	-	-	$$\overset{⌢}{\mathrm{dz}}\mathrm{ɨsɨlib}\left(i\right)\mathrm{әe},\;\overset{⌢}{\mathrm{dz}}\mathrm{ɨsɨ}\;\mathrm{libiәe},\;\overset{⌢}{\mathrm{dʑ}}\mathrm{isɨ}$$
	*Viburnum glomeratum* Maxim.	F	-	-	
Cephalotaxaceae	*Cephalotaxus sinensis* (Rehder & E.H.Wilson) H.L.Li	F	水柏子	Shui Bai Zi	$$\overset{⌢}{\mathrm{tɕ}}\mathrm{eɣu},\;\overset{⌢}{\mathrm{dʑ}}\mathrm{eɣu}$$
Cornaceae	*Cornus japonica* Thunb.	F	荔枝	Li Zhi	
Corylaceae	*Corylus sieboldiana* Blume	F	榛子	Zhen Zi	$$\overset{⌢}{\mathrm{tsɨ}}\mathrm{ga},\;\overset{⌢}{\mathrm{tɕ}}\mathrm{iga}$$
	*Corylus heterophylla* Fisch. ex Besser	F	榛子	Zhen Zi	$$\overset{⌢}{\mathrm{tsɨ}}\mathrm{ga},\;\overset{⌢}{\mathrm{tɕ}}\mathrm{iga}$$
	*Corylus ferox* Wall.	F	榛子	Zhen Zi	$$\overset{⌢}{\mathrm{tsɨ}}\mathrm{ga},\;\overset{⌢}{\mathrm{tɕ}}\mathrm{iga}$$
Dennstaedtiaceae	*Pteridium aquilinum* (L.) Kuhn var. *latiusculum*	L	阳蕨	Yang Jue	$$\mathrm{ɕi}:\overset{⌢}{\mathrm{dʐ}}\mathrm{ala}$$
Eleagnaceae	*Elaeagnus umbellata* Thunb.	F	尖子	Jian Zi	hələ, ʂilə
	*Hippophae rhamnoides* L.	F			$$\mathrm{la}\overset{⌢}{\mathrm{its}}\mathrm{ɨma},\;\overset{⌢}{\mathrm{dʐ}}\mathrm{ɨmɨ}\overset{⌢}{\mathrm{dʐ}}e,\;\mathrm{la}\overset{⌢}{\mathrm{its}}\mathrm{ɨmәgu},\mathrm{laigә}$$
Fabaceae	*Sophora alopecuroides* L.	S(SP)	苦豆	Ku Dou	not recorded
Helwingiaceae	*Helwingia japonica* (Thunb.) F.	L	叶里开花	Ye Li Kai Hua	susaɕi
Juglandaceae	*Juglans cathayensis* Dode	F, FL	野核桃	Ye He Tao	nina, dienanu, dienane = walnut flɔwers[die = walnut tree]
Lamiaceae	*Caryopteris divaricata* Maxim.	L	-	-	nina
	*Mentha haplocalyx* Briq.*	L	薄荷	Bo He	ɕiucә, ɕiәʐә
	*Stachys affinis* Bunge	R	-	-	baʐɨ
Lardizabalaceae	*Decaisnea fargesii* Franch	F	鬼指头	Gui Zhi Tou	$$\overset{⌢}{\mathrm{tɕ}}\mathrm{iar}\overset{⌢}{\mathrm{dɕ}}ә,\;\overset{⌢}{\mathrm{tʂ}}\mathrm{uar}\overset{⌢}{\mathrm{dʐ}}ә$$
	*Akebia trifoliata* (Thunb.) Koidz.	F	-	-	$$\overset{⌢}{\mathrm{dz}}\mathrm{әme}\overset{⌢}{\mathrm{dʐ}}\mathrm{iәu}$$
Liliaceae	*Lilium* sp.	R	大花, 百合	Da Hua, Bai He	ɕiebu
Malvaceae	*Malva verticillata* L.	L	锦葵	Jin Kui	(ng)nagu, nɨnagu, ɨamuda
Meliaceae	*Toona sinensis* (A.Juss.) M.Roem.	L	香椿	Xiang Chun	$$\overset{⌢}{\mathrm{ts}}\mathrm{aiʐɨme}$$
Moraceae	*Morus australis* Poir.	F	野桑子,野桑葚,	Ye Sang Zi, Ye Sang Shen	dusɨbielu, anaʐɨna
Onocleaceae	*Matteucia intermedia* C.Chr.	L	白蕨	Bai Jue	$$\overset{⌢}{\mathrm{dʐ}}\mathrm{ala}$$
	*Matteucia struthiopteris* (L.) Tod.	L	白蕨	Bai Jue	$$\overset{⌢}{\mathrm{dʐ}}\mathrm{ala}$$
Pinaceae	*Pinus armandii* Franch.	S	黄松, 苏木头	Huang Song, Su Mu Tou	tɔm(u)gu, tɔm(u)gə tɔr(ə), tɔʐɨ
Plantaginaceae	*Plantago depressa* Willd.*	L	车前	Che Qian	$$\overset{⌢}{\mathrm{tɕ}}u\overset{⌢}{\mathrm{dʐ}}\mathrm{ia},\;\overset{⌢}{\mathrm{ts}}u\overset{⌢}{\mathrm{dʐ}}\mathrm{iang}\overset{⌢}{\mathrm{dʐ}}i,$$ samɔdɔdeɕi, sɨmatutaɕie
Polygonaceae	*Persicaria alata* (Buch.-Ham.) Nakai*	L	鬼荞	Gui Qiao	$$\mathrm{khiere}\overset{⌢}{\mathrm{dʐ}}\mathrm{ɔulemə},\;\mathrm{tuanle}\overset{⌢}{\mathrm{dʐ}}\mathrm{ɔu},$$ lianmen
Ranunculaceae	*Caltha palustris* L.	L	黄花菜	Huang Hua Cai	$$\mathrm{huamien}\left(\overset{⌢}{\mathrm{ts}}\mathrm{ai}\right),$$ abububa
Rhamnaceae	*Berchemia sinica* C.K.Schneid.	F	提格儿	Ti Ge Er	saü, sawi, saɨu
	*Rhamnus rosthornii* E.Pritz. ex Diels	F	-	-	$$\overset{⌢}{\mathrm{ts}}\mathrm{ɨde}\overset{⌢}{\mathrm{ts}}\mathrm{ɨma},\;\overset{⌢}{\mathrm{ts}}\mathrm{ɨma},\;\overset{⌢}{\mathrm{ts}}\mathrm{ɨdәgɨ}$$
Rosaceae	*Crataegus wilsonii* Sarg.	F	面梨	Mian Li	da:da:
	*Crataegus kansuensis* E.H.Wilson	F	面梨	Mian Li	da:da:
	*Fragaria pentaphylla* Losinsk.	F	瓢子	Piaozi	ɕü
	*Maddenia hypoleuca* Koehne	F	-	-	ɕiana
	*Malus baccata* (L.) Borkh.	F	山定子	Shan Ding Zi	sәmәnia
	*Neillia sinensis* Oliv.	L	茶格	Cha Ge	mahadәʐә, wahadәʐә, wahadәr, mahuɔtu
	*Prunus davidiana* Franch.	F	野毛桃	Ye Mao Tao	ti:, ting, kuaʐә, kuebu, kuɔʐuɔ, kuumɔ
	*Prunus salicina* Lindl	F	苦李子, 野李子	Ku Li Zi, Ye Li Zi	$$\overset{⌢}{\mathrm{dʐ}}\mathrm{ile},\;\overset{⌢}{\mathrm{dz}}\mathrm{ilә}$$
	*Prunus tomentosa* Thunb.	F	-	-	nesɨ
	*Pyrus xerophila* T.T.Yu	F	野酸梨	Ye Suan Li	ɕieʐɨ, ɕiedɔ, $\overset{⌢}{\mathrm{tɕ}}\mathrm{aʐә}$ ɕiɔ:, ɕialә(ng)
	*Rosa brunonii* Lindl.	F	刺格	Ci Ge	$$\begin{array}{l} \overset{⌢}{\mathrm{dʐ}}\mathrm{ie}\overset{⌢}{\mathrm{dʐ}}ɨ\overset{⌢}{\mathrm{ts}}\mathrm{ɨma},\;\overset{⌢}{\mathrm{ts}}\mathrm{ɨmadudu},\;\\ \overset{⌢}{\mathrm{ts}}\mathrm{ɨmadɔdɔ},\;\overset{⌢}{\mathrm{ts}}\mathrm{ɨmate},\;\mathrm{ɕidie}\;\overset{⌢}{\mathrm{dʑ}}\mathrm{iu},\\ \overset{⌢}{\mathrm{dʑ}}\mathrm{ia}\overset{⌢}{\mathrm{ts}}\mathrm{ɨma},\;\overset{⌢}{\mathrm{ts}}ɨ\overset{⌢}{\mathrm{tɕ}}\mathrm{imagu},\;\overset{⌢}{\mathrm{dʐ}}i\overset{⌢}{\mathrm{dʐ}}i \end{array}$$
	*Rubus amabilis* Focke	F	红帽子	Hong Mao Zi	ʐәna, ʐɨna, $\overset{⌢}{\mathrm{dʐ}}\mathrm{eɕiehɔlɔ},$ ʐɨna mɨngʐɨ, ʐɨna ɕiʐu
	*Rubus mesogaeus* Focke	F	黑帽子	Hei Mao Zi	ʐәna, ʐɨna
	*Rubus thibetanus* Franch	F	黑帽子	Hei Mao Zi	ʐәna, ʐɨna, $\overset{⌢}{\mathrm{dʐ}}\mathrm{uәr},$ duheni, ɕiehele, wunihebie
	*Rubus xanthocarpus* Bureau & Franch.	F	黄帽子	Huang Mao Zi	$$\overset{⌢}{\mathrm{dʐ}}\mathrm{uɔ}\left(\overset{⌢}{\mathrm{ts}}\mathrm{ɨma}\right),$$ diuhɨn, diuheni, $$\overset{⌢}{\mathrm{dʐ}}u\left(l\right)e,\;\mathrm{ʐɨna}\overset{⌢}{\mathrm{tɕ}}i$$
	*Sorbus xanthoneura* Rehder	F	黄脉花楸	Huang Mai Hua Qiu	duɔsi(ɕieduɔ), $\overset{⌢}{\mathrm{dʐ}}\mathrm{umaɕiaɔ},\;\overset{⌢}{\mathrm{dʐ}}\mathrm{umaɕieda}$
Rutaceae	*Zanthoxylum bungeanum* Maxim.	L, F	椒芽, 花椒	Jiao Ya, Hua Jiao	ɨime
Salicaceae	*Populus davidiana* Dode	FL	杨树花	Yang Shu Hua	-
	*Salix* spp.	FL	柳花	Liu Hua	ɨanilɔ
Santalaceae	*Buckleya henryi* Diels	F	-		$$\overset{⌢}{\mathrm{dʐ}}\mathrm{әma}$$
Saxifragaceae	*Rodgersia aesculifolia* Batalin**	R	鬼灯擎	Gui Deng Qing	hamahaʐɨ
Schisandraceae	*Schisandra sphenanthera* Rehder & E.H.Wilson	F	五味子	Wu Wei Zi	wi:, wәi
Staphyleaceae	*Staphylea holocarpa* Hemsl.	L	亮子尖	Liang Zi Jian	zәmәduɕiә, $\overset{⌢}{\mathrm{dʐ}}\mathrm{amuduɕie},\;\overset{⌢}{\mathrm{dʐ}}\mathrm{amuduɕie}$ ɕiegie, $$\mathrm{liang}\overset{⌢}{\mathrm{dz}}ɨ,\mathrm{ruanma}$$
Thelypteridaceae	*Pseudocyclosorus subochthoides* (Ching) Ching	L	白蕨	Bai Jue	$$\overset{⌢}{\mathrm{dʐ}}\mathrm{ala}$$
Urticaceae	*Urtica fissa* E.Pritz. ex Diels*	L	荨麻	Qian Ma	sana [mainly], $\mathrm{sapa}\overset{⌢}{\mathrm{dʐ}}a$ [rarely]
Vitaceae	*Vitis piasezkii* Maxim.	F	野葡萄	Ye Pu Tao	$$\mathrm{zɨmuguan}\overset{⌢}{\mathrm{dʐ}}u,\mathrm{zɨmugu}\overset{⌢}{\mathrm{dʐ}}u,\mathrm{zɨmugu}\overset{⌢}{\mathrm{dʐ}}ɨ,\mathrm{ɨemuguɔ}\overset{⌢}{\mathrm{dʐ}}\mathrm{uɔ}$$

Plant parts: L – young leaves, buds or other aerial parts; F – fruits; FL – inflorescences; R – roots, rhizomes etc.; S – seeds; SP – used as spice.

*controversial food plants, nowadays used by very few informants; regarded by most people as inedible or unattractive but usually widely used as food for pigs.

**used only as famine food in 1959–61.

^$^the accent always falls on the last syllable of the word.

**Table 3:** Wild mushrooms collected for food mentioned by at least two informants

**Species**	**Local Chinese name**	**Local Chinese name (pinyin)**	**Local Tibetan name (pronunciation according to IPA)**
*Grifola umbellata* (Pers.) Pilát	猪苓花	Zhu Ling Hua	$\overset{⌢}{\mathrm{dʐ}}\mathrm{ulinmɔ},\overset{⌢}{\mathrm{dʐ}}\mathrm{ulinghua},\overset{⌢}{\mathrm{dʐ}}\mathrm{ulinghәmә}$
*Morchella* spp. (mainly *M. angusticeps* Peck)	羊肚菌	Yang Du Jun	$\mathrm{iangdu}\overset{⌢}{\mathrm{dʐ}}\mathrm{in},$ hәmәʂә, $\mathrm{kuitɔ}\overset{⌢}{\mathrm{dʐ}}\mathrm{ün}$
*Auricularia* spp. (mainly *A. auricula-judae* (Bull.) Quél.)	野木耳	Ye Mu Er	ainɔu
“a red capped mushroom”	红蘑菇	Hong Mo Gu	hәmә, hәmәhә

On average, 16.2 edible taxa were listed per interview (median – 16). The most listed category of wild foods was green vegetables (mean – 8.7 species, median – 9 species), but fruits were listed nearly as frequently (mean – 6.9, median – 6). Other category lists were very short: flowers (mean – 0.2, median – 0), underground edible parts (mean – 0.3, median – 0) and mushrooms (mean – 1.5, – median 1).

In group interviews we obtained only slightly more species per list (e.g. fruits – 6.4 species in individual ones, 6.9 in group ones; green vegetables – 8.3 and 9.2 respectively). The difference between individual and group interviews is small, probably due to the fact that some of the best informants were interviewed separately.

Wild vegetables are usually boiled and/or fried and served as side-dishes (*cai*). Wild fruits are collected mainly by children and eaten raw, they are not stored for further use. Mushrooms are usually fried. During the 1959–61 famine *Sinacalia tangutica* tubers (one of the commonest plants in the roadsides) were widely used as emergency food and they are well remembered, although no one eats them nowadays.

**Figure 8:**
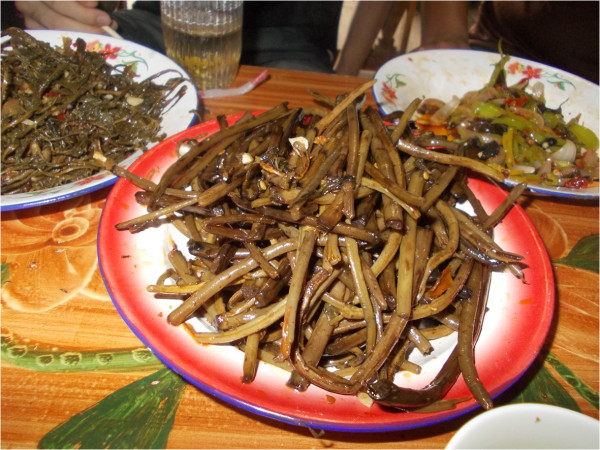
Lacto-fermented stalks of bracken fronds, served fried with chilli and pieces of bacon.

**Figure 9:**
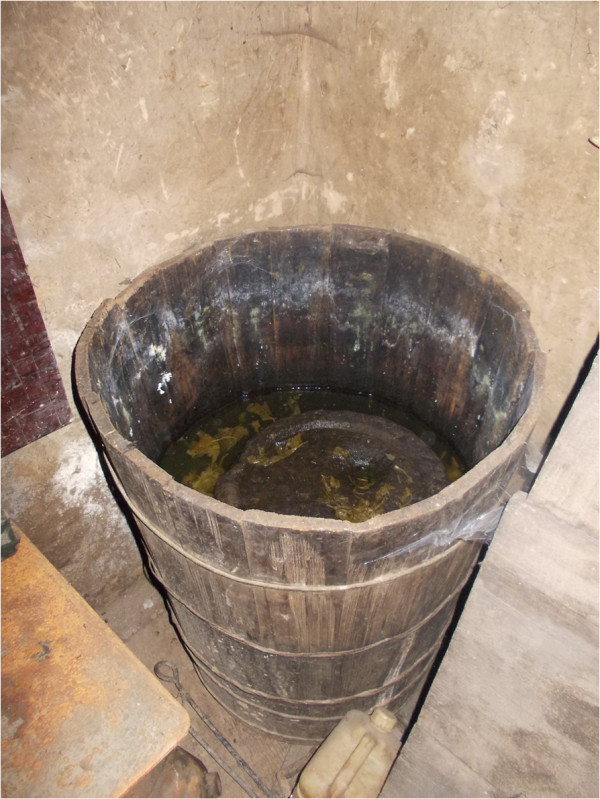
**A barrel with lacto-fermented shoots of *Helwingia japonica.***

**Figure 10:**
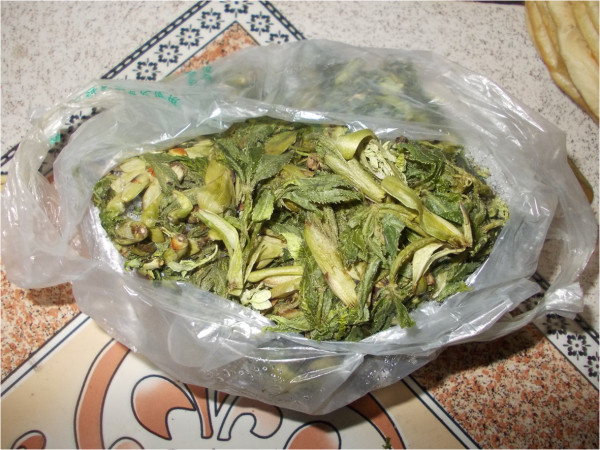
**Freezing has recently become an alternative to lacto-fermenting or drying wild vegetables: frozen *Eleuterococcus *** **leaves.**

Practically all families dry wild vegetables for further use, and many of them lacto-ferment wild vegetables in wooden barrels (around half of the families) while some have recently started freezing wild vegetables for winters in electric refrigerators (Figures 8, 9 and 10).

The most widely used wild vegetables are: *Eleuterococcus* spp., ferns - called collectively *zhala* (pinyin pronunciation), *Helwingia japonica, Aralia chinensis, Allium victorialis, Pteridium aquilinum, Ixeris chinensis*, *Thlaspi arvense* and *Chenopodium album* (Table 1)*.* The first four of these taxa (*Eleuterococcus* spp., ferns, *Helwingia japonica* and *Aralia chinensis*) are also traded throughout the county and are mentioned even in the Zhouqu county annals as important sources of income for the locals [29].

It is interesting that some wild greens, which are very common species in the valley and are eaten in other parts of China, e.g. wild amaranths *Amaranthus* spp. and nettles *Urtica* spp. [6,26,27], are almost entirely ignored, regarded as inedible by most of the population, and eaten by very few individuals.

The most commonly eaten fruits are: wild pears *Pyrus xerophila*, wild plums *Prunus salicina*, *Berchemia sinica*, blackberries *Rubus* spp. (black fruited) and *Eleagnus umbellata.* The large number species of fruits reported in this study, compared to the central part of the Qinling Mts. [26,27], probably stems from the different landscape. Here, there is an abundance of natural hedgerows growing on steep banks dividing the field terraces, providing large masses of edible fruits near homesteads.

The number of wild taxa eaten in the studied valley is relatively large compared to most studies around the world. However, compared to the northern slope of the Qinling, the list is shorter, in spite of the similar methodology applied and similar research effort involved [26,27]. This may stem from the combined influences of two factors:

1. the local flora is relatively poorer here (the rainfall is lower, forest understory is poorer and the forest vegetation is more transformed by humans)

2. the local population is less interested in using all the potentially edible wild vegetables, for example the extremely abundant *Amaranthus* and *Urtica* are not utilized by the majority of the population.

In our previous papers [26,27] from northern Qinling we noted that local people seem to value forest wild greens over the ruderal taxa. This is even more visible in this part of the Qinling, where the gathering efforts are nearly exclusively oriented towards forest greens. Local people venture six km hikes up steep slopes in order to collect ferns, marsh marigolds *Caltha palustris* and bittercress *Cardamine macrophylla*, and do not bother to collect annual wild greens growing around their homesteads!

The culinary use of *Caltha palustris* is very interesting. In its raw state marsh marigold is a highly toxic plant, due to the presence of protoanemonin, and has not been eaten recently. We only have historical reports about its use in northern Europe, mainly from Estonia [39], and from the United States (as potherb – [40], or flower buds as caper substitutes – [41]). Some plant dictionaries have also recorded the edibility of *Caltha* leaves and roots, e.g. Kluk’s *Dykcyonarz Roślinny* from eighteenth century Poland [42]. Protoanemonin is broken down by drying and long cooking [43,44]. In the study area, the plant is either dried and then used like other wild greens, or it is lacto-fermented after the initial blanching. The dried specimens of marsh marigolds we ate had no bitterness even prior to cooking. Further studies are needed to establish if the local population of marsh marigolds is a low-protoanemonin form easier to prepare as food than the European *Caltha.* For example, one of the authors’ (ŁŁ) unpublished experiments show that *C. palustris* from Poland needs one to two hours of cooking to get rid of the bitterness caused by protoanemonin. Some species of the related genus *Ranunculus* have been eaten in Europe after only short cooking, as they are not as bitter as European *Caltha,* e.g. *R. repens* L. in Belarus and Poland [45], *R. neapolitanus* Ten. in Croatia [46], *Ranunculus muricatus* L. in Herzegovina [46] and *R. ficaria* L. in Slovakia [47], Ukraine [48], Romania and Hungary [49] and Italy [50].

It is interesting that the list of wild edible fruits is slightly longer than that of wild greens, as the reverse was usually the case in wild food studies in East Asia. The structure of the edible species list (similar number of wild greens and fruits) is very similar to that recorded among the Tibetans in Shangri-La [12] where 80 wild vegetable species and 78 edible fruit species were recorded. It is also more reminiscent of other regions of the world such as Africa [51,52] and India [53,54], where wild greens are used widely but not as many species as in the Qinling Mountains and other regions of East Asia, where the local biodiversity enables a choice from many species of wild vegetables.

Similarly to the Shaanxi part of the Qinling mountains, the number of fungi taxa collected is low. There were only four taxa mentioned by more than four informants, whereas as many as 153 species of edible mushrooms were identified in the area [34]. The two most commonly collected taxa (*Grifola* and *Morchella*) do not have original Tibetan names, but names borrowed from Chinese, which suggests that it was the Chinese who introduced their use. In contrast to the mushrooms, there is high consensus on wild vegetable and fruit names and all of them possess local Tibetan names.

The high level of bilinguality as far as plant names are concerned is worth emphasizing. Although bi- and trilinguality is very common in cultural edge areas (Zhouqu county has been a Tibetan-Chinese ethnic borderland for centuries), a nearly full bilinguality concerning plant names is a much rarer phenomenon. When we asked our informants why they know nearly all the local Chinese names of edible plants (although they speak only Tibetan in their villages) they explained that they need them when they talk about plants with people from the neighbouring Han villages. This is a proof that fortunately wild edible plants still constitute an important topic of conversation for the villagers and they maintain a high cultural status.

## Conclusions

The number of wild taxa eaten in the studied valley is relatively large compared to most studies of edible plants from throughout the world. However, compared to the northern slope of the Qinling, the list is shorter, in spite of the similar methodology applied and similar research effort involved. The culinary use of the toxic *Caltha palustris* is worth emphasizing.

## Competing interests

The authors state that they have no competing interests.

## Authors’ contributions

YK organized the expedition, led the interviews, identified most taxa and drafted parts of the text. ŁŁ wrote the first version of the article and processed the data. YK, ŁŁ, JK, FW, JH took active part in the interviewing process in the field, voucher specimen preparation and data processing, and read the final version of the manuscript. QG took part in the interviewing process, organized group meetings and helped to design the study. All authors read and approved the final manuscript.
